# Supplementary material for: Molecular detection of Coxiella burnetii infection in aborted samples of domestic ruminants in Iran
Source: PLoS One. 2021 Apr 14;16(4):e0250116. doi: 10.1371/journal.pone.0250116 (PMC8046214; doi:10.1371/journal.pone.0250116)
Supplement: S2 Table — (DOCX) [file pone.0250116.s002.docx]

S2 Table. Data of Cow abortions sampling.

| Province | Farm code | No. of Sample | No. of Positive |
| --- | --- | --- | --- |
| Tehran | A | 4 | 1 |
|  | B | 5 | 2 |
|  | C | 3 | 0 |
|  | D | 14 | 6 |
|  | E | 3 | 0 |
|  | F | 4 | 0 |
|  | G | 4 | 0 |
| Ardabil | A | 1 | 0 |
| East-Azarbaijan | A | 1 | 0 |
| Hamadan | A | 1 | 0 |
| Alborz | A | 3 | 0 |
|  | B | 3 | 1 |
