# Supplementary material for: Molecular detection of Coxiella burnetii infection in aborted samples of domestic ruminants in Iran
Source: PLoS One. 2021 Apr 14;16(4):e0250116. doi: 10.1371/journal.pone.0250116 (PMC8046214; doi:10.1371/journal.pone.0250116)
Supplement: Attachment — Submitted filename: Reviewer Response.docx [file pone.0250116.s003.docx]

**Author’s Response to Reviewers Comment**

Note: All changes showed in Revised Article with Changes Highlighted uploaded file and made by Track Change.

**Reviewer 1#**

Reviewer #1: The manuscript by Mobarez et al., describes a survey of 146 tissue samples from spontaneously-aborted cattle, sheep, and goats for the presence of Coxiella burnetii by RT-PCR, using IS1111 as a target. Based on previous reports, results of the study are not surprising. Moreover, originality of the work is nominal and rests solely on the fact that Iranian livestock abortion samples were tested.

**Reviewer** **comment 1:** There are numerous English grammar and syntax errors in the text. My suggestions to the authors are itemized below.

**Author’s Response 1:** Thanks for your comments. All recommended grammatical comments were considered. Manuscript was revised by a native English reviewer.

**Reviewer comment 2:** How did the authors establish that ≤ 36 cycles was the RT-PCR Ct value for positivity?

**Author’s Response 2:** Based on our previous setting, tenfold-serial dilutions of DNA from control positive (*C. burnetii* Nine Mile strain) were prepared and tested by RT-PCR according to recommended reference (“Real-Time PCR with Serum Samples Is Indispensable for Early Diagnosis of Acute Q Fever, Clin Vaccine Immunol. 2010 Feb; 17(2): 286–290”). We defined the limit of detection of our assay as the lowest concentration of genome equivalents. A CT value of <36.0 was considered positive.

**Reviewer comment 3:** How many RT-PCR analyses were done per sample? Were any technical replicates performed? Did the authors check any PCR products on agarose gels to ensure that the amplicon size was correct for the target?

**Author’s Response 3:** The positive samples tested three times by RT-PCR. Also, we checked PCR products of positive samples on 2% agarose gels (70 bp). Finally, the samples that had a positive first test and the next two tests were positive and also had the acceptable amplicon size on the agarose gel were considered as true positive.

**Reviewer comment 4:** The Discussion section tends to be redundant with the Results section, and repeats much of the same information.

**Author’s Response 4:** Thanks. Revised.

**Reviewer comment 5:** Please use standard journal abbreviations in the References section**.**

**Author’s Response 5:** Done.

**Reviewer 2#**

Reviewer #2: The authors present a study on ‘Molecular detection of Coxiella burnetii in aborted samples of livestock in Iran’. The objective of this study was to report the prevalence of C. burnetii in convenient samples from clinical aborted ruminants. There is novelty on the present study, considering the little information of this disease in Iran and implications for public health. Although the paper needs considerable review.

**Reviewer comment 1:** I found the manuscript difficult to read and to understand, due to the sentence structure, and incorrect use of terminology. Before to address the scientific content, I advise the authors to have the manuscript reviewed for English and veterinary/ epidemiology terminology.

**Author’s Response 1:** Manuscript was revised by a native English reviewer.

**Reviewer comment 2:** Title: The study is focused on ruminant samples (not in livestock in general), the title should reflect that.

**Author’s Response 2:** Title was revised.

**Reviewer comment 3:** Abstract: The abstract used interchangeable samples from livestock, but samples are from ruminants, the reader is misled by the title and abstract (the same throughout the manuscript). The study was conducted using convenience sampling submitted to the national lab (this should be described in the abstract). The conclusions are very general and open-ended. Actually, without knowing the overall prevalence in ruminants, reporting only the clinical cases/ samples submitted for diagnosis may increase the apparent prevalence.

**Author’s Response 3:** Thanks. Revised.

Abortion samples are not routinely referred and submitted to national laboratory by Veterinary organization and Clinical Veterinarians. All samples of this study were sampled only for this study. None of the samples were systematically referred to our laboratory.

**Reviewer comment 4:** Introduction: I found the introduction confusing, lacking of flow between the paragraphs, as the authors cite epidemiology status in animals and humans back and forward without connecting thoughts/ sentences. Also, the use of inappropriate terminology is observed in this section as for example:

Line 35: ‘chronic Q fever is extremely serious and dangerous’ in what aspect? To whom?

Line 46: ‘dairy factory workers’ – dairy farms? Processing plants?

Line 47: ‘during livestock calving’ – delivery? Calving used for cows (kidding for goats, etc)

Line 48: ‘harsh environmental conditions’- do you mean extreme environmental conditions?

Line 51: ‘In recent years, C. burnetii has become one of the most important causes of abortion in livestock’s ‘– Worldwide? or specific country? What do you mean by ‘livestock’s’.

Although Q fever can affect many domestic animals, the effects on abortion are most likely to appear in small ruminants in a naïve population. Please be specific.

Line 55: ‘childbirth infected animals’ – please review.

Line 57’ ‘Abortion by wind’- do you mean aborted products can be easily aerosolized and transmitted by wind? Please review.

**Author’s Response 4:** Thanks. The introduction section of manuscript was revised based on these comments.

**Reviewer comment 5:** Methods: This study uses a convenience sample from ‘cooperation of clinical veterinarians as well as the Iranian Veterinary- Organization’ – how many samples from each source? Please make a reference that this is convenience sample from clinical cases of aborted ruminants. There were different aborted specimens tested, presented how many of each type tested. Do you expect differential specificity/ sensitivity based on the sample type? If yes, what are the impacts on your findings (add a section for limitations in the discussion section). For the PCR methods, was this protocol developed by your lab, if not please cite the protocol source.

Line 72:’ All livestock abortions were spontaneous’. Again, here should be ruminant abortions, by spontaneous do you mean clinical? What were the age of abortion for the tested samples?

**Author’s Response 5:** The Methods section of manuscript was revised based on these comments.

Abortion samples are not routinely referred and submitted to national laboratory by Veterinary organization and Clinical Veterinarians. None of the samples were systematically referred to our laboratory. The role of the veterinary organization was to coordinate and introduce us to the farmers. All samples of this study were sampled only for this study. Finally, sampling was done by ourselves or our representative.

In most cases, only one specimen type was taken from aborted fetus samples. In cases where several specimens were taken from the aborted fetus samples, the PCR test result was completely consistent. Due to the low number of specimens ranging from an aborted fetus cannot talk properly about the sensitivity and specificity of different specimens. Specimens type was added results section.

Also, our RT-PCR test was done according to recommended reference which cited and available in PCR section of methods (“Real-Time PCR with Serum Samples Is Indispensable for Early Diagnosis of Acute Q Fever, Clin Vaccine Immunol. 2010 Feb; 17(2): 286–290”).

Unfortunately, one of the limitations of our work was the failure to record the age of the aborted fetus, which we noticed at the end of the study. This point was added to discussion section as a limitation.

**Reviewer comment 6:** Results: The information in Tables 1 and 2 can be summarized in one table. Provide the total of positive samples and denominator for each %. e.g. line 112: 24,32%. Table legends are incomplete (add date of the study and location/ country). 95% CI should be added to the results.

**Author’s Response 6:** Thanks. Results section was revised based on comments.

**Reviewer comment 7:** Discussion: The authors made an attempt to emphasis the public health and animal health impact of the findings in aborted samples. Therefore, the need for surveillance programs to be implemented in Iran. However, one of the big pitfalls of the study is the sample size and convenience sampling, without knowing how representative are those samples of the herd prevalence, external validity, those claims are difficult to assess. One of the novel aspects of this study is that the authors used PCR comparing to most of studies used seroprevalence. How different are those studies: studied population, diagnostic testing ( Se, Sp), samples tested ( clinical versus surveillance), etc? The authors briefly described the prevalence in other studies/ countries but do not provide a justification for such numeric difference. This should be explored in the discussion section.

**Author’s Response 7:** Discussion section was revised based on comments.

Reviewer comment about small samples size and sampling in this study is correct. We added this point is a limitation in discussion section.

All similar studies, which listed in the discussion section, used convenience sampling like our study. Samples of these studies belonged to investigation studies and clinical samples. Also, wWe mentioned that “One of the reasons for the high prevalence in our study was the using of a much more sensitive method (Real-time PCR) for detection of *C. burnetii*. Other reasons include different study areas and increased prevalence over time.”.

**Reviewer comment 8:** Pitfalls are not addressed in the manuscript.

**Author’s Response 8:**  Limitations were added to discussion section.
