# Supplementary material for: Molecular detection of Coxiella burnetii infection in aborted samples of domestic ruminants in Iran
Source: PLoS One. 2021 Apr 14;16(4):e0250116. doi: 10.1371/journal.pone.0250116 (PMC8046214; doi:10.1371/journal.pone.0250116)
Supplement: Attachment — Submitted filename: Reviewer Response2.docx [file pone.0250116.s004.docx]

**Author’s Response to Reviewers Comment**

Note: All changes showed in Revised Article with Changes Highlighted uploaded file and made by Track Change.

**Reviewer 1#**

Reviewer #1: The manuscript by Mobarez et al., describes a survey of 146 tissue samples from spontaneously-aborted cattle, sheep, and goats for the presence of Coxiella burnetii by RT-PCR, using IS1111 as a target. This is a revised version of the manuscript, but it still contains numerous items that need to be addressed.

**Reviewer** **comment 1:** The authors’ responses to Reviewer 1 comments 2 and 3 (i.e., information regarding how they established that ≤ 36 cycles was the RT-PCR Ct value for positivity, how many RT-PCR analyses were done per sample, replicates, etc., were not added to the revised manuscript. Please add this information to the materials and methods or at least cite a reference(s) so readers know how the PCR was done and verified.

**Author’s Response 1:** These points were added to manuscript. Also, refence of RT-PCR Ct value was added to manuscript.

**Reviewer comment 2:** The authors have transposed the headings on the actual S1 Table and S2 Table (they are correct on lines 176 and 177). They should read “S1 Table. Results of sheep abortion sampling” And “S2 Table. Results of cow abortion sampling.

**Author’s Response 2:** Thanks. Revised.

**Reviewer comment 3:** In certain instances, the authors use average values rounded to the hundredth place, while in others they round to the tenth place. Please round up all figures to the tenth place throughout the paper for consistency. For example, line 20 should read 24.7% (not 24.66%).

**Author’s Response 3:** All figures were Revised based on Reviewer comment.

**Reviewer comment 4:** Lines 21 and 107- The average value reported is not correct. 20 of 94 sheep samples is 21.3%, not 21.78% as shown.

**Author’s Response 4:** Thanks, revised.

**Reviewer comment 5:** Lines 100-102- The authors state that “94 samples…were from sheep abortions (S1 Table)”. However, if you look at S1 Table (after correcting for the heading transposition), there are 89 samples in column 3, not 94 as stated. Please clarify.

**Author’s Response 5:** 94 samples is correct. We forgot to add 5 samples of West Azerbaijan province in S1 Table. S1 Table was revised. Thanks for your attention.

**Reviewer comment 6:** Lines 102-104- The authors state that “46 samples…were from cattle abortions (S2 Table)”. However, if you look at S2 Table (after correcting for the heading transposition), there are 48 samples in column 3, not 46 as stated. Please clarify.

**Author’s Response 6:** 46 sample is correct. S2 Table was revised.

**Reviewer comment 7:** Minor editorials (English, grammar, etc.) comments:

7. Line 17- should read “…to investigate the occurrence of C. burnetii…”: Done.

8. Line 18- should read “…from cattle, sheep and goats…” (cattle and sheep are plural in English) : Done.

9. Lines 19 and 86- should read “…targeting the IS1111…”: Done.

10. Line 25- should read “…paid to the role…”: Done.

11. Line 26- should read “…veterinary organizations.” :Done.

12. Indent for new paragraphs at lines 35, 51, 61, 77, 106, 112, 151 and 162: Done.

13. Line 33- should read “…16S rRNA …belongs to…”: Done.

14. Line 41- change “animals” to animals’ or animal: Done.

15. Line 47- should read “…febrile illness with…and is asymptomatic…”: Done.

16. Line 52- delete “the”: Done.

17. Line 53- should read “…are more likely to appear in small…”: Done.

18. Line 56- replace “dose” with “density”, as dose refers to load given to animal/human: Done.

19. Line 58- delete “transmitted” (redundant with previous line): Done.

20. Lines 108 and 113- should read “The highest prevalence…”: Done.

21. Line 115- should read “…goat-aborted…” and “…four of the samples…”: Done.

22. Line 127- should read “…be seriously considered in cases…”: Done.

23. Line 128- should read “…to humans regarded.” and “…fever, yet it is still…”: Done.

24. Line 131- should read “…in the human health care…”: Done.

25. Line 139- replace “most” with “highest” :Done.

26. Line 140- should read “…Iran, wherein the …”: Done.

27. Line 144 and 158- replace “using” with “use” :Done.

28. Line 154- replace “with” with “in” :Done.

29. Line 155- should read “…countries, the prevalence…”: Done.

30. Line 162-163- should read “…to make prevalence comparisons.” Also replace “large” with “greater” :Done.

31. Line 164- should read “It is also recommended that non-convenience…”: Done.

32. Lines 166-167- should read “…fetuses, can inform the epidemiology…”: Done.

33. Line 192- do the authors mean large-animal veterinarians? Also- replace “helps” with “help : Done.

34. Lines 143-144 and 157-158 are redundant. “One of the reasons…”: Done.

35. Table 1 legend shown with the actual table (lines 257-258) is not the same as that as shown on lines 118-119 the text. It should read "...sheep and cattle abortion..." for consistency's sake. :Done.

**Author’s Response 7:** All done. Thank you very much for your valuable comments.

**Reviewer 2#**

Reviewer #2: Title: ‘Molecular detection of Coxiella burnetii in aborted samples of livestock in Iran’.

The authors addressed the main concerns of the reviewers, although, the discussion could be improved. The authors added most of the information requested by the reviewers, but there is some redundancy regarding the results, moreover comparison with other studies is very descriptive without a link or comparison between the present findings, and lacking on interpretation and potential reasons for similar (or not) findings (see examples below). I have some minor comments regarding the terminology/ grammar as the following.

**Reviewer comment 1:** Minor changes:

Line 18: remove ‘s , ‘ change to from cattle, sheep and goats’: Done.

Line 26 : ‘veterinary organization’, should be ‘veterinary organizations’? : Done.

Line 55:‘of transmission in the environment’, change to contamination of the environment.: Done.

Line 60: ‘ in the transmission of the disease’ , change to ‘ in the transmission risk to humans’: Done.

Line 73 ‘cotyledons’, placenta cotyledons: Done.

Line 76: ‘…. Was done under a BSL2 biological safety cabinet’, maybe change to ‘…was done under biosafety level 2 (BSL2) laboratory settings’ : Done.

Line 124: ‘ C. bunetti in domestic animal abortions’, change to ‘… in ruminant abortions’: Done.

Lines 127-129. I am not sure what the authors are trying to convey here. Please rephrase this sentence: Done.

Lines 153-136: It seems it is missing a linking sentence. Suggestion: “Actually Q fever is one of the causes of endocarditis in humans, it was reported that about of 31 % ……”: Done.

Lines 140: remove ‘…., so that the….’ , change too ‘. Molecular prevalence….’: Done.

Lines 145: ‘…like our study.’, change to ‘similar to our study’. Here is one of the examples that I think the discussion is very simplistic. Although the authors added this sentence as response to the previous reviewer, they haven’t expanded on limitations of prevalence studies using convenience sampling, are those representatives of the true prevalence? are those studies comparable? etc.: Done.

Lines 149- 150. Another example of simplistic discussion, why we should give more attention to abortion in sheep due to C. burnetti.: Done.

Lines 167: ‘… better judgements.’’, change to ‘…inform better…’ : Done.

Line 173: ranchers or farmers? Ranchers.

**Author’s Response 1:** All done. Thank you very much for your valuable comments.
